# College Courant, New Haven

**Published:** 1872-11

**Authors:** 


***The College Courant,***

Recently placed under new editorial management, brings to the support of its columns increased aid of material from both English and Foreign resources, considerably more interesting and important to educated readers generally, than is contained in any other single publication now accessible to the American public.

Among those who contribute are—

President Chadbourne, of Williams College; Ex-President Woolsey, of Yale; President F. A. P. Barnard, of Columbia; President A. D. White, of Cornell University; Professor Alex. Winchell, Michigan University; Prof. Moses Coit Tyler, of Michigan University; President Porter, of Yale; Prof. C. H. Hitchcock, of Dartmouth; Prof. John Bascom, of Williams; Prof. James D. Dana, of Yale; Prof. A. E. Dolbear, of Bethany; Prof. J. B. Sewall, of Bowdoin; Prof. J. P. Lacroix, of Ohio Wesleyan University; Prof. Oliver Marcy, of Northwestern University.

And many others, including eminent college professors and the best literary talent in the country.

It contains educational news from all parts of the world; full intelligence and criticisms of new books; the best items of intelligence, and discussion from English and Foreign journals; editorials on a variety of topics interesting to educated persons, and carefully prepared notes. The following are recent notices of the Courant:

"The College Courant comes to us this week announcing a change of editorship. It is evident that an earnest and scholarly editor has assumed the reins." —*The Independent.*

"The College Courant promises to increase largely its importance and value under the new editorship. Its chief care will be to mark the development of the system of higher education, and to reflect as completely as may be the progressing changes in the fields of letters, science and art. It addresses itself, therefore, to all people of culture, and especially to the nation's educators, journalists, teachers, and the college community."—*The New York Evening Mail.*

"It is far the best paper devoted to the subject of education generally, and to University training in all its phases. The new measures proposed will tend to make it a necessary aid to all personally interested in the work of academic training. We can heartily commend it to our readers."—*Zion's Herald, Boston.*

"There is no doubt that the new editorial management will do all that fine scholarship, high purpose and indefatigable industry can do. We expect to see the Courant take its place among the few journals which no man or woman of thorough education can afford to neglect."—*The Index, Toledo, Ohio.*

The subscription price of *The Courant* is $4.00 a year, or it will be sent for three months, on trial, for $1.00. Single copies, ten cents. For sale by all newsdealers.

Remit by Post Office money order, check or draft, to publishers "*College Courant*," New Haven, Conn.

